# Are long-lasting insecticide-treated bednets and water filters cost-effective tools for delaying HIV disease progression in Kenya?

**DOI:** 10.3402/gha.v8.27695

**Published:** 2015-06-10

**Authors:** Stéphane Verguet, James G. Kahn, Elliot Marseille, Aliya Jiwani, Eli Kern, Judd L. Walson

**Affiliations:** 1 Department of Global Health and Population, Harvard T.H. Chan School of Public Health, Boston, MA, USA; 2 Philip R. Lee Institute for Health Policy Studies, University of California, San Francisco, CA, USA; 3 Global Health Sciences, University of California, San Francisco, CA, USA; 4 Department of Epidemiology and Biostatistics, University of California, San Francisco, CA, USA; 5 Health Strategies International, Oakland, CA, USA; 6 Health Strategies International, Arlington, VA, USA; 7 Assessment, Policy Development & Evaluation, Public Health-Seattle & King County, WA, USA; 8 Department of Global Health, University of Washington, Seattle,, WA, USA; 9 Department of Medicine, University of Washington, Seattle, WA, USA; 10 Department of Pediatrics, University of Washington, Seattle, WA, USA; 11 Department of Epidemiology, University of Washington, Seattle, WA, USA; 12 Centre for Clinical Research, Kenya Medical Research Institute, Nairobi, Kenya

**Keywords:** cost savings, insecticide-treated bednets, water filtration, HIV disease progression, antiretroviral therapy, malaria, diarrhea, Kenya, sub-Saharan Africa

## Abstract

**Background:**

Co-infection with malaria and other infectious diseases has been shown to increase viral load and accelerate HIV disease progression. A recent study in Kenya demonstrated that providing long-lasting insecticide-treated bednets (LLIN) and water filters (WF) to HIV-positive adults with CD4 >350 cells/mm^3^ significantly reduced HIV progression.

**Design:**

We conducted a cost analysis to estimate the potential net financial savings gained by delaying HIV progression and increasing the time to antiretroviral therapy (ART) eligibility through delivering LLIN and WF to 10% of HIV-positive adults with CD4 >350 cells/mm^3^ in Kenya.

**Results:**

Given a 3-year duration of intervention benefit, intervention unit cost of US$32 and patient-year ART cost of US$757 (2011 US$), over the lifetime of ART patients, in Kenya, we estimated the intervention could yield a return on investment (ROI) of 11 (95% uncertainty range [UR]: 5–23), based on a cost of about US$2 million and savings in ART costs of about US$26 million (95% UR: 8–50) (discounted at 3%). Our findings were subjected to a number of sensitivity analyses. Of note, deferral of time to ART eligibility could potentially result in 3,000 new HIV infections not averted by ART and thus decrease ART cost savings to US$14 million, decreasing the ROI to 6.

**Conclusions:**

Provision of LLIN and WF could be a cost-saving and practical method to defer time to ART eligibility in the context of highly resource-constrained environments experiencing donor fatigue for HIV/AIDS programs.

More than two-thirds of the world's individuals living with HIV reside in sub-Saharan Africa. About half of these 25 million individuals do not yet meet World Health Organization (WHO) ‘priority’ eligibility criteria to initiate antiretroviral therapy (ART), specified as having a CD4 count of 350 cells/mm^3^ or less, and among those that do meet these ‘priority’ criteria, about 70% are receiving ART (1, 2).

International development assistance for health has grown dramatically (from $5.7 billion in 1990 to $28.1 billion in 2012) (3), and with the support of initiatives such as the Global Fund to Fight AIDS, Tuberculosis and Malaria and the United States President's Emergency Plan for AIDS Relief (PEPFAR), funding specifically for HIV/AIDS has also increased (from $0.7 billion in 2000 to $6.8 billion in 2010) (3). As a result, the number of individuals in low- and middle-income countries receiving ART has grown from an estimated 300,000 people in 2002 to approximately 10 million in 2012 (1).

Despite these massive investments, the projected costs of supporting ART in the coming decades will rapidly outpace projected funding. The aids2031 Costs and Financing Project (4) estimates global funding needs ranging from $400 to $700 billion for 2009–2031, with funding needs for low- and middle-income countries nearing $40 billion annually by 2031 – three times the current level (5, 6). Therefore, the cost of universal, life-long access to ART appears unsustainable given the current funding climate (7). In addition, development assistance for HIV/AIDS has flattened after years of steep rises, leading to an even greater need for implementing cost-effective interventions in highly resource-constrained environments (8).

Interventions that can delay HIV disease progression can moderate the growing demand for ART and result in considerable ART-related savings. Such interventions can free financial and human resources to be directed both toward HIV prevention programs and treatment for individuals with advanced disease. A recent study in Kenya demonstrated that providing a long-lasting insecticide-treated bednet (LLIN) and a point-of-use water filter (WF) to HIV-positive adults not yet on ART delayed progression of HIV disease (9). Specifically, after 2 years of follow-up and after controlling for baseline CD4 count, those individuals having received LLIN and WF were 27% less likely to reach the endpoint of a CD4 count <350 cells/mm^3^ (HR: 0.73, *p*=0.02) than those not having received LLIN and WF; rate of CD4 decline was also significantly less in the intervention group (−54 cells/mm^3^/year vs. −70 cells/mm^3^/year, *p*=0.03) (9). In addition, the provision of LLIN and WF, in the context of this study and when part of an integrated prevention campaign including HIV counseling and testing, has been shown to be highly cost-effective (10, 11).

In addition to the cost savings associated with a deferral of time to ART eligibility, these interventions directly target several other important infectious diseases endemic to sub-Saharan Africa, including diarrhea and malaria (12, 13). Across sub-Saharan Africa in 2010, diarrhea and malaria accounted for more than 80,000 and 190,000 deaths among adults aged 15–49 years, respectively (14). Regardless of their effect on HIV progression, LLIN and WF are essential tools that would benefit all individuals living in settings where diarrhea and malaria are common, with greatest benefit among individuals living with HIV who are at increased risk for diarrhea and malaria (15–19).

In settings with endemic malaria and non-potable water, common in sub-Saharan African countries, LLIN and WF are valuable interventions for most households. In particular, LLIN and WF given to HIV-positive individuals awaiting ART eligibility can prevent malaria and diarrhea infections among these individuals and their household members. Moreover, the provision of these interventions can delay HIV progression in this population, moderate the growing demand for ART, and subsequently decrease ART-related costs by deferring time to ART eligibility.

In this article, we used the findings of the Kenya study (9) to estimate the prospective ART-related cost savings when HIV-positive individuals not yet eligible for ART [CD4 count >350 cells/mm^3^ according to WHO ‘priority’ eligibility criteria (2)] are given LLIN and WF, in Kenya. This article does not argue for prioritization of LLIN-WF to HIV-positive individuals as opposed to the general population, or against ART initiation for those individuals with CD4 count >350 cells/mm^3^. Rather, due to the growing demand for ART and the realities of funding constraints, we acknowledge that most individuals will not start ART before reaching a CD4 of 350. We focus our analysis accordingly on the financial benefits of providing LLIN-WF to this population compared with the status quo of no ART treatment before CD4 350.

## Methods

We estimated total costs and ART cost savings for the provision of LLIN and WF to 10%^1^ of HIV-positive adults (15 years and above) awaiting ART eligibility,^2^ aware of their HIV status, in Kenya. The ART cost savings were estimated over a lifetime horizon, over the lifetime of HIV-positive individuals. The mean lifetime on ART was assumed to be 33 years when individuals initiated ART at a CD4 count of 350 cells/mm^3^ [average between a Ugandan estimate (20) and a South African estimate (21)], and it was assumed to be the same for all adults. Our analysis focused on ART cost savings, that is, on how ART costs may be deferred (discounted) in time and bring net financial savings. The costs were discounted using a 3% discount rate consistently with cost-effectiveness guidelines (22, 23). Note that likely small increases in background mortality due to deferral of ART initiation were neglected. The duration of intervention benefit is assumed to be *T*_*int*_=3 years; that is, LLIN and WF are assumed to be effective for 3 years [Vestergaard-Frandsen personal communication; Ref. (24)]. This analysis was performed from the perspective of the healthcare provider. All costs were reported in 2011 US dollars.

### Data

We utilized the findings from the multisite Kenya trial (9). The trial followed two prospective cohorts comprising 589 ART-naïve HIV-1-positive adults from HIV clinics at two sites in Western Kenya. Individuals enrolled in the intervention cohort received a LLIN and a WF. Individuals in the control cohort did not receive a LLIN or a WF. Individuals in the intervention cohort had a mean CD4 count at enrollment of 530 cells/mm^3^ (IQR: 450–670) as opposed to 550 cells/mm^3^ (IQR: 440–690) in the control cohort. After 2 years, individuals in the intervention cohort were 27% less likely to reach the endpoint of a CD4 count < 350 cells/mm^3^, after controlling for baseline CD4 count.

The subsequent relative effectiveness, *D*_*eff*_=27%, of delaying HIV progression was retained as the base case for Kenya. The effectiveness might be indeed higher/smaller in each province of Kenya. However, in lieu of this simpler generalization, we had no empirical data to inform a potential extrapolation of effectiveness to each Kenyan province. We addressed limitations pertaining to this assumption in the sensitivity analyses, where a substantial (±50%) variation in the key input parameters was implemented to clearly identify the main drivers of the results.

The number of adults that were HIV-positive and the number of adults that were on ART was sourced from UNAIDS (1). The LLIN-WF intervention cost was set at *c*_*int*_=US$32 per person per campaign, using recently published estimates (25) for an integrated campaign implemented in Kenya (a different setting than the trial setting). This estimate is the projected unit cost of a scaled-up replication of the Kenya campaign as would be applied to HIV-positive individuals who are not yet eligible for ART. The US$32 per person cost includes US$6 for the malaria intervention (LLIN and staff training) and US$16 for the diarrhea intervention (WF and staff training). Though we assumed LLIN-WF to be provided to HIV-positive individuals aware of their HIV status, the additional costs for HIV testing (test kits, counseling, condoms, and CD4 testing) were included, that is, US$10 (25). The annual cost per patient for ART and associated HIV care was assumed to be *c*_*ART*_=US$757. The figure of $757 per person-year of ART is the authors’ construction as this data was not available for Kenya. This is an average cost figure derived from the cost figures reported for low-income countries (Benin, Ethiopia, Haiti, Uganda) from a recent review article (26), combined with recently published cost estimates for Ethiopia and Uganda from two PEPFAR-supported country programs (27), and from cost estimates for rural Uganda and the average cost for 45 sites in Zambia, extracted from two additional publications (28, 29). The cost figures derived from Gálarraga et al. (26) were reported in 2009 US$ after foreign currency conversion using average annual exchange rates and adjustment for inflation using the US consumer price index (CPI) by the authors. We further adjusted these figures to 2011 US$ using the US CPI. The cost figures from Menzies et al. (27), Marseille et al. (28) and Marseille et al. (29) all reported in US$ were similarly adjusted to 2011 US$ using the US CPI.

All costs used were reported in 2011 US$. We varied the cost of ART per person-year in the sensitivity analyses.

### Approach

The LLIN-WF intervention targets *N*_*adults*_ adults. The total cost of the intervention would be *TC*_*int*_ *=N*_*adults*_ *c*_*int*_, and the total savings in ART care would be *TC*_*ART,A*_. *TC*_*ART,A*_ depends on the following inputs: *N*_*adults*_, *c*_*ART*_, *l*_*ART*_, *D*_*eff*_, *v*_1_, *T*_*int*_, *CD4*_*st*_, and *r*. Notably *l*_*ART*_ is the lifetime on ART; *D*_*eff*_ is the effectiveness; *v*_1_ is the rate of decline of CD4 count for the group of HIV-positive individuals not yet on ART not receiving LLIN-WF, that is, −70 cells/mm^3^/year (9); *T*_*int*_ is the effectiveness duration (3 years here); *CD4*_*st*_ is the CD4 count in the HIV-positive adult population at time of LLIN-WF provision; and *r* is the annual discount rate [chosen to be 3% (22, 23)]. A ‘return on investment’ (ROI) for the intervention can then be defined as follows: $ROI=\frac{TC_{\mathrm{ART},A}}{TC_{Int}}$. Further details on how *TC*_*ART, A*_ was derived are given in the Supplementary file (Section 1).

First, we present the ROI for the base case and examine how it varies with key parameters (*c*_*ART*_, *D*_*eff*_, *l*_*ART*_, *CD4*_*st*_). Second, we report on Kenya-specific results. All key parameter base case values used in the analysis are listed in Table 1.

**Table 1:** Key base case inputs used in the analysis of the intervention providing bednets and water filters to HIV-positive adults to delay HIV disease progression in Kenya

Input	Value	Source
Duration of intervention effectiveness *T*_*int*_	3 years	Vestergaard-Frandsen personal communication; Clasen et al. (24)
Intervention cost per person *c*_*int*_	$32	Kahn et al. (25)
Annual ART cost per patient *c*_*ART*_	$757	Gálarraga et al. (26); Menzies et al. (27); Marseille et al. (28); Marseille et al. (29)
Relative effectiveness in delaying HIV progression *D*_*eff*_	27%	Walson et al. (9)
Fraction of HIV-positive adults with CD4 > 350 cells/mm^3^ receiving LLIN-WF	10%	Authors’ assumption
Mean CD4 count of HIV-positive individuals at time of LLIN-WF provision *CD4*_*st*_	500 cells/mm^3^	Authors' assumption based on: Larson et al. (32); Lugada et al. (33); Lessells et al. (34); Govindasamy et al. (35); Akinbami et al. (36); Williams et al. (37)
Lifetime on ART *l*_*ART*_	33 years	Mills et al. (20); Johnson et al. (21)
Estimated number of adults aged 15 years and above in need of ART	680,000	UNAIDS (1)
ART coverage (%)	81	UNAIDS (1)
HIV prevalence (15 years and above) (%)	6.1	UNAIDS (1)

ART, antiretroviral therapy; LLIN, long-lasting insecticide-treated bednet; WF, water filter.

### Sensitivity analysis

We assessed the robustness of our findings using both univariate and multivariate sensitivity analysis. First, a multivariate sensitivity analysis was conducted using Monte Carlo simulations (*n*=100,000 trials) where all key parameters (*D*_*eff*_, *c*_*ART*_, *c*_*int*_, % of HIV-positive individuals with CD4>350 receiving the intervention, *l*_*ART*_, *CD4*_*st*_) were varied simultaneously. Parameter uncertainty was included through sampling *n* values for each parameter to which was assigned either a Gamma or Beta distribution built on each input's mean and standard deviation (30), resulting in *n* samples. Finally, extracting the 2.5 and 97.5 percentiles allowed the determination of 95% uncertainty ranges (URs), which are reported with the results. Further details are given in the Supplementary file (Section 3.1).

Second, univariate sensitivity analyses were performed including: 1) to seek the smallest *c*_*ART*_ value for which ROI=1 and 2) to seek the smallest *D*_*eff*_ value for which ROI=1.

In addition, we estimated the number of new HIV infections not averted by ART which could be attributed to deferring time to ART eligibility for those who received LLIN and WF, assuming 0.05 infections per person-year not on ART (31) (an extreme upper bound given that individuals are in HIV care and receive HIV counseling and condoms). Expected lifetime ART costs corresponding to these additional infections were deducted from the estimated ART cost savings of the campaign, in order to highlight the worst-case scenario. Further detail is given in the Supplementary file (Section 3.2).

All analyses were conducted using R (www.r-project.org) and Mathematica (Wolfram Research, Inc., Mathematica, Version 8.0, Champaign, IL, 2010).

## Results

Given a 3-year duration of intervention benefit, intervention effectiveness of 27%, intervention unit cost of US$32, patient-year cost of ART of US$757, and *CD4*_*st*_=500 cells/mm^3^ (Table 1), we estimated an ROI of 11.1 (95% UR: 4.7–22.7) (Table 2). When effectiveness is assumed at half that found in the Kenya trial (13.5%), the ROI would decrease to 4.7; when effectiveness is 50% higher (40.5%), the ROI would increase to 17.1. When the annual ART cost per patient is assumed at half that previously assumed (US$379), the ROI would decrease to 5.5; when annual ART cost per patient is 50% higher, the ROI would increase to 16.6. When *CD4*_*st*_=400 cells/mm^3^, the ROI would decrease to 3.9; when *CD4*_*st*_=530 cells/mm^3^ (as observed in the trial), the ROI would slightly increase to 11.3. When lifetime on ART is assumed at half that in the base case (16.5 years), the ROI would be 7.1 (Table 2). Finally, ROI>1 until the effectiveness of delaying HIV progression decreases to *D*_*eff*_ =3% and the annual ART cost per patient reaches *c*_*ART*_=US$70. In Kenya, the LLIN-WF intervention would cost about US$2.3 million (95% UR: 1.4–3.5) but would save US$25.5 million (95% UR: 8.0–49.8) (discounted at 3%). From a savings standpoint, the intervention would present net financial savings of US$23.2 million (95% UR: 5.9–47.2).

**Table 2:** Return on investment results for the intervention providing bednets and water filters to HIV-positive adults to delay HIV disease progression in Kenya

Scenario	Return on investment
Base case (*D*_*eff*_=27%; *c*_*int*_=$32; *c*_*ART*_=$757; *CD4*_*st*_=500 cells/mm^3^; *l*_*ART*_=33)	11.1
Effectiveness is halved (*D*_*eff*_=13.5%)	4.7
Effectiveness is 50% higher (*D*_*eff*_=40.5%)	17.1
ART cost is halved (*c*_*ART*_=$379)	5.5
ART cost is 50% higher (*c*_*ART*_=$1136)	16.6
*CD4*_*st*_=400 cells/mm^3^	3.9
*CD4*_*st*_=530 cells/mm^3^	11.3
*l*_*ART*_=16.5 years	7.1
Lifetime ART costs due to new infections not averted by ART included	5.9

ART, antiretroviral therapy; LLIN, long-lasting insecticide-treated bednet; WF, water filter.

### Sensitivity analysis

If intervention coverage is increased (20% of HIV-positive adults with CD4>350 receive LLIN-WF), total ART cost savings would amount to $51 million in Kenya. If intervention coverage is decreased (5%), total ART cost savings would amount to US$13 million in Kenya. When the annual ART cost per patient is set at half that previously assumed (US$379), total ART cost savings would decrease to US$13 million in Kenya.

It is important to recognize that HIV-positive individuals who are not initiated on ART remain a potential source of new infections. Our findings indicate that deferring time to ART eligibility could potentially result in an additional 2,800 new HIV infections not averted by ART in Kenya (Supplementary file, Section 3.2). This could translate to US$11 million (discounted) lifetime ART costs in Kenya, subsequent decreased ART cost savings of US$14 million in Kenya, and a decreased ROI of 5.9 (95% UR: 1.9–13.6) (Table 2), when it is assumed that: 70% of the newly HIV-positive individuals would seek treatment [mean ART coverage for HIV-positive individuals with CD4 >350 cells/mm^3^ is currently 68% in sub-Saharan Africa (1)]; half of these individuals would initiate ART early [leading to a subsequent lifetime on ART of about 33 years, which is the average of a Ugandan estimate of 36 years (20) and a South African estimate of 29 years (21)]; and the remaining half would initiate ART late [leading to a subsequent lifetime on ART of about 11 years (38)]. Further detail is given in the Supplementary file (Section 3.2).

**Fig. 1:**
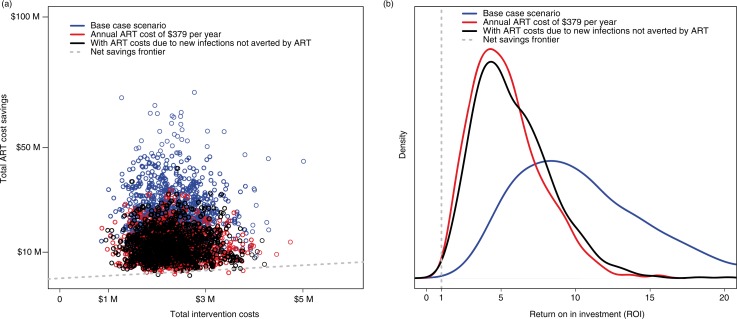
Comparison of the base case scenario with the two scenarios where the annual ART costs are halved to $379 and when lifetime ART costs due to new infections not averted by ART are included. (a) Total ART cost savings versus total intervention costs in 2011 US$ (*n*=1,000 trials extracted from Monte Carlo simulation), and (b) distribution of return on investment (ROI) function (*n*=1,000 trials extracted from Monte Carlo simulation). ART, antiretroviral therapy; *n*=number of simulations. Note: the net savings frontier corresponds to the situation when total ART cost savings equals total intervention costs.

In spite of considerable uncertainty, the LLIN-WF intervention still presented net financial savings even when conservative scenarios were explored: for example, when ART unit cost per patient-year was substantially decreased, and when lifetime ART costs due to potential added new infections not averted were included (Fig. 1).

## Discussion

The provision of LLIN and WF to 10% of HIV-positive adults not yet eligible for ART in sub-Saharan Africa could potentially yield high returns on investment and bring substantial net financial savings due to deferred time to ART eligibility. This finding of substantial savings in Kenya was subjected to both univariate and multivariate sensitivity analyses. It was most sensitive to the effectiveness in delaying HIV progression, the mean CD4 count at time of LLIN-WF provision, and the cost of ART per person-year.

While a LLIN-WF intervention for HIV-positive individuals awaiting ART initiation is an economically attractive intervention, it may not be a favorable option based on other considerations. Studies indicate that persons receiving ART may experience a greater than 90% reduction in HIV transmission to their partners (39–42). Hence, deferring time to ART eligibility may increase the transmission of HIV, and accordingly, the LLIN-WF intervention described in this analysis could result in a number of potential new infections. Second, earlier initiation of ART can also prevent both AIDS- and non-AIDS-related morbidity and mortality (43); and the economic benefits including labor productivity gains of earlier ART initiation can be substantial. Therefore, although the ROI of a LLIN-WF intervention remains favorable, deferring time to ART eligibility may not be acceptable from several standpoints including a public health or clinical standpoint, and the cost analysis offered in this article is only one element among others into the decision-making.

This article does not argue for prioritization of LLIN-WF provision to HIV-positive individuals as opposed to the general population, nor does it prioritize provision of LLIN-WF to HIV-positive individuals as opposed to early initiation of ART, which may provide high returns on health. Rather, it recognizes that given the growing demand for ART and financial resources constraints, early ART initiation or ART initiation for those with CD4 >350 and/or >500 is often unaffordable and unfeasible. Given that reality, provision of LLIN-WF to HIV-positive individuals awaiting ART initiation can bring substantial net financial savings.

There are several limitations to this analysis. First, the results rely heavily on data from two Kenyan studies (9, 25). The respective contributions of LLIN and WF to the effectiveness in deferring ART are unclear, and this effectiveness may well change as the burden of diarrhea/malaria varies from one setting to the next. However, empirical data do not exist to inform a better extrapolation. Our objective in this article was indeed to examine the potential economic impact of distributing LLIN-WF to HIV-positive individuals awaiting ART initiation, in the context of highly resource-constrained settings. Second, since identifying HIV-positive non-ART-eligible individuals would require additional costs, we restricted our analysis to the subset of HIV-positive individuals who are aware of their HIV status. There is a lack of data on the number of HIV-positive individuals who are aware of their HIV status and the proportion of these that could feasibly be reached by a LLIN-WF campaign; as such, we chose a conservative estimate of 10% coverage of HIV-positive individuals with CD4 cell account above 350 cells per mm^3^, and we assumed that only HIV-positive ART non-eligible individuals were receiving LLIN-WF. Furthermore, a more detailed assessment with use of a dynamic model of HIV transmission would help incorporate secondary HIV infections resulting from added HIV infections not averted by ART. Finally, this article does not estimate the intervention health benefits (e.g. malaria, diarrhea, HIV infections averted) in order to maximize population health per dollar spent; rather, we indicate that the intervention brings net financial savings when uniquely considering the HIV dimension. LLINs and WFs are essential interventions for diarrhea and malaria and should be made available, especially to pregnant women and children, regardless of their effect in delaying HIV disease progression.

The provision of LLIN and WF may be a cost-effective and practical method for ART programs to defer ART eligibility in the context of highly resource-constrained environments and donor fatigue for HIV/AIDS programs. Such an intervention could free financial and human resources to be allocated toward HIV prevention programs and maximizing the number of individuals in critical need of ART treatment, while focusing on deferring time to ART eligibility among those not yet meeting WHO ‘priority’ eligibility criteria (2). Integrated prevention programs could thus contribute to the long-term viability of ART scale-up in sub-Saharan Africa. Future work should examine the feasibility of using limited HIV budgets to pay for LLIN-WF when current funds are insufficient to initiate new HIV patients on ART, and of using malaria budgets to provide LLIN-WF in the context of deferring time to ART eligibility.

Countries and donors must define a policy framework which can maximize positive synergies between HIV programs and health systems (44). When broad health goals cannot be met by health systems, high-impact interventions provided through integrated programs are often used as an interim measure. ‘Selective primary health care’, a strategy focused on maternal and child health, is one example (45). Inherent in the integrated delivery platform is the potential for it to be diversified, taking advantage of economies of scale and scope and combining partnerships to improve efficiency and effectiveness (46). For example, in sub-Saharan Africa, LLINs have begun to be distributed via mass immunization campaigns (47, 48), which has enabled a scale-up in use (49). LLIN-WF delivery could be similarly assimilated by the existing HIV program infrastructure, including stewardship and governance, financing, planning, delivery, monitoring and evaluation, and demand creation (50, 51).

Development assistance for HIV/AIDS has been shown to strengthen health systems in some countries but can negatively impact health services where human resources are limited (52). International donors and policy makers should therefore weigh the costs and benefits of integration efforts: leveraging HIV program infrastructure to launch integrated prevention programs shows much promise, but where such infrastructure is nascent or fragile, it may undermine HIV programs themselves (53). Integrated prevention programs can be an effective and cost-saving strategy to deliver interventions and to identify individuals with HIV or other diseases, including tuberculosis and non-communicable diseases. The scale and rate of their rollout should be evaluated in the context of a thorough understanding of the strengths and weaknesses of host health systems, as well as of the commitments of local leaderships, international donors, and the health workforce to particular delivery platforms (51).

## Authors’ contributions

SV, JGK and JLW initiated the study. SV, AJ, EK and EM obtained the data for the analysis. SV, JGK and JLW coordinated the research and did the analysis. EM, EK and AJ reviewed the paper and provided suggestions. SV wrote the manuscript. JGK, JLW, EK and AJ edited the manuscript. All authors read and approved the final version of the manuscript.

## Conflict of interest and funding

JLW and SV received financial support from Vestergaard-Frandsen (VF), the manufacturer of the bednets and water filters used in the Kenya study, for travel to the 2012 International AIDS Conference, and JLW is receiving compensation for advising VF on the development of scientific advisory board. JGK was funded by NIDA DA15612 and JGK, EM and AJ were contracted by VF for another analysis. VF had no role in the study design, data collection, data interpretation, or writing of the manuscript.

## Supplementary Material

Are long-lasting insecticide-treated bednets and water filters cost-effective tools for delaying HIV disease progression in Kenya?

## Footnotes

^1^Ten percent is meant to capture a conservative coverage estimate achievable by country health systems.

## Footnotes

^2^ART eligibility is defined here as a CD4 count of 350 cells/mm^3^ or less: these are the individuals to prioritize for ART initiation, as indicated by WHO [2].
