# Supplementary material for: Are long-lasting insecticide-treated bednets and water filters cost-effective tools for delaying HIV disease progression in Kenya?
Source: Glob Health Action. 2015 Jun 10;8:10.3402/gha.v8.27695. doi: 10.3402/gha.v8.27695 (PMC4463495; doi:10.3402/gha.v8.27695)
Supplement: Are long-lasting insecticide-treated bednets and water filters cost-effective tools for delaying HIV disease progression in Kenya? [file GHA-8-27695-s001.pdf]

Supplementary data

Are long-lasting insecticide-treated bednets and water filters cost-effective tools for delaying HIV disease progression in Kenya?

by

Stéphane Verguet, James G. Kahn, Elliot Marseille, Aliya Jiwani, Eli Kern,

Judd L. Walson

1. Mathematical derivations

We present here the mathematical derivations used for the estimation of antiretroviral therapy (ART)-related cost savings due to deferred time to ART eligibility calculated over a timeframe including lifetime ART costs.

Consider v_1 the rate of decline of CD4 count (in cells per mm^3 per year) for the group of HIV-infected adults not yet on ART *not* receiving a long-lasting insecticide-treated bednet (LLIN) and a water filter (WF), and v_2 for the group receiving LLIN and WF. We denote T_{int} the effectiveness duration of the intervention (i.e. 3 years) and D_{eff} the delay effectiveness. Hence: $D_{eff} = 1 - \frac{v_2}{v_1}$.

Consider **individuals *not* receiving LLIN and WF**. Lifetime ART costs for one of these individuals is given by:

$$c_1 = c_{ART} \sum_{k=t_1}^{t_1+l_{ART}} \frac{1}{(1+r)^k}, \quad (1)$$

with:

$$t_1 = (CD4_{st} - 350)/v_1. \quad (2)$$

t_1 is the time at which the individual initiates ART i.e. when her CD4 count reaches 350 cells/mm^3. $CD4_{st}$ is the CD4 count at which the individual would be given LLIN and WF, r is the discount rate (3%), c_{ART} is the unit cost of one patient-year on ART, l_{ART} is the mean lifetime on ART assumed to be 33 years when individuals initiate ART at a CD4 count of 350 cells/mm^3: this figure is an average between a Ugandan estimate (36 years) [1] and a South African estimate (29 years) [2]. When $CD4_{st} = 500$ cells/mm^3, $v_1 = 70$ cells/mm^3/year: $t_1 = 2.1$ years, and $c_1 = \$15{,}480$ for $c_{ART} = \$757$.

Consider **individuals receiving LLIN and WF**. Lifetime ART costs for one of these individuals is given by:

$$c_2 = c_{ART} \sum_{k=t_2}^{t_2+l_{ART}} \frac{1}{(1+r)^k}, \quad (3)$$

with:

$$t_2 = T_{int} + (CD4_{st} - 350 - v_2 T_{int})/v_1 \text{ if } CD4_{st} > v_2 T_{int} + 350,$$

$$= (CD4_{st} - 350)/v_2 \text{ if } CD4_{st} < v_2 T_{int} + 350, \quad (4)$$

t_2 is the time at which the individual initiates ART i.e. when her CD4 count reaches 350 cells/mm^3. When $CD4_{st} = 500$ cells/mm^3, $v_1 = 70$ cells/mm^3/year: $t_2 = 2.7$ years, and $c_2 = \$15{,}110$ for $c_{ART} = \$757$.

Subsequently, for *N* individuals whose CD4 > 350, we encounter the following ART cost savings (due to discounting):

$$TC_{ART,A} = N(c_1 - c_2), \quad (5)$$

and the following return on investment (ROI):

$$ROI = \frac{TC_{ART,A}}{Nc_{int}} = \frac{c_1 - c_2}{c_{int}}. \quad (6)$$

The ROI can further be expressed as:

$$ROI = \frac{c_{ART}}{c_{int}r}(1+r)^{-\left(\frac{CD4_{st}-350}{v_1}+l_{ART}+D_{eff}T_{int}\right)}((r+1)(1+r)^{l_{ART}}-1)(-1 + (1+r)^{D_{eff}T_{int}})$$

if $CD4_{st} > v_2T_{int} + 350$

$$= \frac{c_{ART}}{c_{int}r}(1+r)^{-\left(\frac{CD4_{st}-350}{v_1}+l_{ART}\right)}((r+1)(1+r)^{l_{ART}}-1)(1-(1+r)^{\frac{CD4_{st}-350}{v_1}+\frac{D_{eff}}{D_{eff}-1}})$$

if $CD4_{st} < v_2T_{int} + 350$ (7)

2. Country-specific inputs and results

2.1. Country-specific inputs

Table S1 presents country-specific parameters used in our calculations. These include: prevalence of HIV (15 years and above) [3]; coverage of ART (among those with CD4 < 350 cells/mm^3) [3], estimated population of adult (15 year-olds and above) HIV-positives with CD4 > 350 cells/mm^3, estimated population of adult (15 years and above) HIV-positives with CD4 > 350 cells/mm^3 receiving LLIN-WF. In particular, from Table S1,

we estimate that there are a total of about 70,000 recipients of the LLIN-WF intervention in Kenya.

Table S1. HIV-related Kenya parameters: prevalence of HIV, coverage of antiretroviral therapy (ART), estimated HIV-positive population with CD4 > 350 cells/mm^3, and estimated HIV-positive population with CD4 > 350 cells/mm^3 receiving LLIN-WF.

**HIV prevalence (%)**	**ART coverage (%)**	**Estimated HIV-positive population with CD4 > 350 cells/mm^3**	**Estimated HIV-positive population with CD4 >350 cells/mm^3 receiving LLIN-WF**
6.1	81	720,000	72,000

LLIN, long-lasting insecticide-treated bednet; WF, water filter.

3. Sensitivity analyses

3.1. Multivariate sensitivity analysis

We conducted a Monte Carlo multivariate sensitivity analysis to estimate aggregate uncertainty from key inputs. Parameters were given values using probability distributions (details are given in Table S2).

Table S2. Probability distributions of the key inputs for the intervention providing bednets and water filters to HIV-positive adults to delay HIV disease progression in Kenya.

Input	Parameters
Intervention cost c_{int} ($)	Gamma with mean = 32, SD = 5
Annual ART cost per patient c_{ART} ($)	Gamma with mean = 757, SD = 129
Relative effectiveness in delaying HIV progression D_{eff}	Beta with mean = 0.27 SD = 0.08
Fraction of untreated HIV-infected individuals with CD4 > 350 receiving LLIN-WF	Beta with mean = 0.10, SD = 0.02
Lifetime on ART l_{ART} (years)	Gamma with mean = 33, SD = 6
Mean CD4 count of HIV-infected individuals at time of LLIN-WF provision $CD4_{st}$ (cells/mm^3)	Gamma with mean = 500, SD = 25

SD, standard deviation; ART, antiretroviral therapy; LLIN, long-lasting insecticide-treated bednet; WF, water filter.

3.2. Lifetime ART costs associated with HIV infections due to delayed ART

The total number of HIV infections that can be attributed to deferred ART corresponds to the number of years of life not on ART multiplied by the number of infections per year not on ART (0.05 [4]). Given the fact that individuals are in HIV care and have received HIV counseling and condoms, 0.05 is a high value which will lead to the estimation of a worst-case scenario. If the total number of individuals receiving

LLIN-WF is N, the total number of years of life 'not on ART' or 'shifted' (discounted at r = 3%) is given by:

$$YL_{ART,A} = N[\sum_{k=t_1}^{t_1+l_{ART}} \frac{1}{(1+r)^k} - \sum_{k=t_2}^{t_2+l_{ART}} \frac{1}{(1+r)^k}], \quad (10)$$

as derived from (1-4), which is estimated at about 56,000 for Kenya, leading to about 2,800 new infections not averted by ART.

The annual cost for a person on ART is assumed to be c_{ART} = \$757. The figure of \$757 per person-year of ART is the authors' construction and is an average cost figure derived from the cost figures reported for low-income countries (Benin, Ethiopia, Haiti, Uganda) from a recent review article [5], combined with recently published cost estimates for Ethiopia and Uganda from two PEPFAR-supported country programs [6], and from cost estimates for rural Uganda and the average cost for 45 sites in Zambia, extracted from two additional publications [7,8]. Further details on the authors' construction can be obtained upon request.

The expected total lifetime cost of providing ART to one individual, LC_{ART}, is discounted to present value using an annual discount rate r = 3%. An HIV-infected individual is assumed to initiate ART with a probability of 0.70 (ART coverage for individuals with CD4 > 350 cells/mm^3 is currently 68% in sub-Saharan Africa [3]). Conditional on ART initiation, a ½ chance is assumed for early ART initiation (when CD4 count reaches 350 cells per mm^3, at about 4.8 years since infection [9,10]) and the subsequent lifetime on ART is assumed to be 33 years. This figure is an average between a Ugandan estimate (36 years) [1] and a South African estimate (29 years) [2]. The remaining ½ chance is assumed for late ART initiation (when CD4 is below 200

cells/mm^3, at about 9.4 years since infection [9,10]) and the subsequent lifetime on ART is assumed to be 11 years. This figure is derived from an analysis which synthesized data from 34 HIV treatment cohorts in sub-Saharan Africa and accounted for loss to follow-up [11]. Hence:

$$LC_{ART} = 0.70\, c_{ART}[\tfrac{1}{2}\textstyle\sum_{k=4.8}^{37.8}(1+r)^{-k} + \tfrac{1}{2}\sum_{k=9.4}^{20.4}(1+r)^{-k}] \qquad (11)$$

We estimated: LC_{ART} = \$7,060. This could translate to \$11 million (discounted) lifetime ART costs and subsequent decreased ART cost savings of \$14 million for the intervention. Evidently, these costs are sensitive to the assumptions retained, including the fraction of HIV-infected individuals that initiate ART and the fraction of those individuals that initiate ART late as opposed to early, and the subsequent duration of ART that is affected by drop-out rates and loss to follow-up.

References

1. Mills EJ, Bakanda C, Birungi J, Chan K, Ford N, et al. Life expectancy of persons receiving combination antiretroviral therapy in low-income countries: a cohort analysis from Uganda. Annals of Internal Medicine 2011; 155(4):209-16.
2. Johnson L, Mossong J, Dorrington R, Schomaker M, Hoffmann C, et al. Life expectancies of HIV-positive adults receiving antiretroviral treatment in South Africa. Presented at the Actuarial Society of South Africa's 2012 Convention 16-17 October 2012, Cape Town International Convention Centre. [http://www.actuarialsocietyconvention.org.za/assets/pdf/papers/Leigh%20Johnson%20-%20LIFE%20EXPECTANCIES%20OF%20HIV-POSITIVE%20ADULTS.pdf]
3. UNAIDS. Global report: UNAIDS report on the global AIDS epidemic, 2013. Geneva, Switzerland: UNAIDS; 2013.
4. Granich RM, Gilks CF, Dye C, De Cock K, Williams BG. Universal voluntary HIV testing with immediate antiretroviral therapy as a strategy for elimination of HIV transmission: a mathematical model. Lancet 2009; 373:48-57.

5. Gálarraga O, Wirtz VJ, Figueroa-Lara A, Santa-Ana-Tellez Y, Coulibaly. Unit costs for delivery of antiretroviral treatment and prevention of mother-to-child transmission of HIB: a systematic review for low- and middle-income countries. Pharmacoeconomics 2011; 29(7):579-99.
6. Menzies NA, Berruti AA, Berzon R, Filler S, Ferris R, et al. The cost of providing comprehensive HIV treatment in PEPFAR-supported programs. AIDS 2011; 25(14):1753-60.
7. Marseille E, Kahn JG, Pitter C, Bunnell R, Epalatai W, et al. The cost-effectiveness of home-based provision of antiretroviral therapy in rural Uganda. Applied Health Economics and Health Policy 2009; 7(4):229-243.
8. Marseille E, Giganti MJ, Mwango A, Chisembele-Taylor A, Mulenga L, et al. Taking ART to scale: determinants of the cost and cost-effectiveness of antiretroviral therapy in 45 clinical sites in Zambia. PLoS ONE 2012; 7(12): e51993.
9. Eligibility for ART in lower income countries collaboration. Duration from seroconversion to eligibility for antiretroviral therapy and from ART eligibility to death in adult HIV-infected patients from low and middle-income countries: collaborative analysis of prospective studies. Sexually Transmitted Infections 2008; 84:i31-36.
10. Hallett TB, Baeten JM, Heffron R, Barnabas R, de Bruyn G, et al. Optimal uses of antiretrovirals for prevention in HIV-1 serodiscordant heterosexual couples in South Africa: a modeling study. PLoS Medicine 2011; 8(11):e1001123.
11. Verguet S, Lim SS, Murray CJL, Gakidou E, Salomon JA. Incorporating loss to follow-up in estimates of survival among HIV-infected individuals in sub-Saharan Africa enrolled in antiretroviral therapy programs. Journal of Infectious Diseases 2013; 207(1):72-79.
